# Dual-use artificial intelligence and biology: upstream risk-benefit reviews

**DOI:** 10.3389/fmicb.2026.1832974

**Published:** 2026-06-01

**Authors:** Moritz S. Hanke, Shrestha Rath, Anita Cicero, Thomas V. Inglesby, Jaspreet Pannu

**Affiliations:** 1 Center for Health Security, Bloomberg School of Public Health, Johns Hopkins University, Baltimore, MD, United States; 2 Bloomberg School of Public Health, Johns Hopkins University, Baltimore, MD, United States

**Keywords:** artificial intelligence, biological AI models, biorisk management, biosecurity, dual-use, pandemic risk management, responsible innovation and governance, risk–benefit

## Abstract

Biological AI models (BAIMs) are advancing rapidly and hold substantial promise. Yet, these models raise dual-use concerns, particularly regarding capabilities that could enhance pathogens with pandemic potential. Current risk mitigation discussions for BAIMs are concentrated in the post-development stage, focusing on evaluations and safeguards, after a model has been trained. We argue that upstream, pre-development risk–benefit review (RBR) is a necessary, missing component of effective BAIM governance. The broad range of models, their capabilities, and purpose-built applications, as well as a majority-academic developer community, make this approach feasible and realistic. We propose a review framework with the following five components and discuss key characteristics of each component: (1) review trigger criteria that determine whether a model should undergo an RBR. The review trigger criteria are (A) a model is reasonably anticipated to possess capabilities of concern or (B) it will be trained on sensitive pathogen data classified under the Biosecurity Data Levels (BDL) system; structured risk (2) and benefit (3) reviews using qualitative and quantitative criteria; (4) integration of risk and benefit scores into a composite assessment; and (5) proportionate risk mitigation recommendations. We expect that such reviews (2 through 5) would apply to only a small fraction of BAIMs and would benefit responsible developers by establishing clear expectations at the outset of model development. We discuss how such a framework could be implemented broadly across academic institutions, commercial developers, and federal, philanthropic, and private funding bodies, and address limitations such as the subjectivity of risk assessments. RBR for BAIMs remains nascent and will require expert-driven working groups to define capabilities of concern, establish clear review criteria, and assess risk mitigation efficacy. RBRs are a promising conceptual approach for BAIM risk management and should be pioneered, refined, and vetted through real-world application with model developers.

## Introduction

### Dual-use risks from biological AI models

Artificial intelligence (AI) for biology is advancing rapidly and promises remarkable benefits for human health, agriculture, and the environment. Biological AI models (BAIMs), i.e., models trained on substantial quantities of non-natural-language biological data such as biological sequence, structure, function, or imaging data, have emerged as a class of models with a wide range of capabilities.

While BAIM advances have yielded impressive results, such as generating functional proteins that do not exist in nature (Hayes et al., 2025; Merchant et al., 2026), current models also possess significant dual-use concerns (Pannu et al., 2024; Zhang et al., 2025a). For instance, the same model that can generate high-affinity antibody binders against influenza haemagglutinin for therapeutic purposes can equally be misused to optimize binding between viral surface proteins and host cell receptors (Bennett et al., 2025). The generation of novel bacteriophage genomes supported by genomic language models raised concerns about using similar approaches for genomes of smaller viruses with pandemic potential (Johnson, 2025; King et al., 2025).

BAIMs allow users to compress trial-and-error laboratory research into ever more accurate *in silico* predictions of the outcomes (Biswas et al., 2021; Ingraham et al., 2023). For instance, where host-susceptibility and serial-passing experiments in animals were previously required to determine the host range and effects of mutations in viruses with pandemic potential, BAIMs can increasingly predict these (Hie et al., 2021; Gonzalez-Isunza et al., 2023; Thadani et al., 2023; Gurev et al., 2025). Also, BAIMs are being integrated with AI agents and AI-enabled autonomous laboratories to form rapid, iterative design-build-test-learn (DBTL) cycles (Smith et al., 2026). This amplifies dual-use concerns about integrating laboratory results to optimize the harmful properties of pathogens with pandemic potential (Rapp et al., 2024; Wang et al., 2026).

The misuse potential of BAIMs has repeatedly been publicly highlighted by scientists. Baker and Church noted that “computational protein design […] is vulnerable to misuse and the production of dangerous biological agents” (Baker and Church, 2024). In a public statement, nearly 200 leading protein design scientists acknowledged the misuse risks of AI-enabled biodesign and agreed to measures for responsible development and security risk mitigation (Responsible AI x Biodesign, 2024). The 2025 Asilomar entreaty, endorsed by world-leading scientists in AI and biology, stated that “AI tools can be exploited to generate synthetic toxins, engineer pathogens, or bypass regulatory oversight” (Bromberg et al., 2025).

### Current risk reduction landscape for biological AI models

Legally binding requirements or standardized frameworks to reduce misuse risks from BAIMs in academia or the private sector do not currently exist. A report analyzing more than 1,100 BAIMs found that fewer than 1.5% of models had any safeguards against misuse (Atanasov et al., 2026). Recent findings demonstrated how BAIMs can be used to circumvent existing biosecurity safeguards like nucleic acid synthesis screening software by designing functional homologs. However, they also demonstrated that synthesis screening software could be patched accordingly and that the functional homologs did not have sufficient activity when tested in the laboratory (Wittmann et al., 2025; Ikonomova et al., 2026). Still, with the rapid advances in AI-enabled protein design, the authors stress the need to develop safeguarding strategies and evaluations to track model misuse risk. When safeguards for BAIMs are implemented, this generally occurs in an *ad hoc* fashion, with no external testing or validation to confirm the efficacy of the chosen safeguards. While we commend some responsible actors for pioneering safeguards, mitigating misuse risk from BAIMs is a complex task, necessitating concrete tools and institutionalized practices that support researchers in responsible practices.

Debate about risk reduction for BAIMs has mostly centered around evaluations and built-in safeguards (Fan et al., 2025; Gurev et al., 2025; Wang et al., 2025; Wei et al., 2025). Notably, both of these only become applicable after a model has been developed. After model development, implementing technical safeguards becomes more resource-intensive, as existing capabilities need to be modified or monitored computationally, or model access needs to be regulated. Also, commercial and academic incentives pressure model developers to publish as soon as possible, with limited capacity for safeguards and risk evaluations. Many BAIMs are published without peer review, which could surface security concerns, and are often released with open weights (Atanasov et al., 2026). Once released with open weights, models are irrevocably distributed, with no way to implement *post hoc* safeguards or monitor misuse-relevant usage. Additionally, it is possible to further modify open-weight BAIMs to improve their performance on misuse-relevant tasks, for instance, via fine-tuning on viral data (Black et al., 2025). Ultimately, structured security assessments remain largely absent from the early model development stages. Current risk reduction efforts focus on the post-development stage, where effectiveness and scalability may be limited.

### Learning from existing dual-use risk reduction frameworks

Approaches to reduce misuse risks from BAIMs can draw on decades of careful thinking, consensus-building, and policy development by scientists, biosecurity practitioners, and policymakers on how to manage biological dual-use risks. Many frameworks acknowledge that it is infeasible to prevent all conceivable biological harm and instead prioritize the most consequential ones. This has led many in the field of biological risk reduction to prioritize identifying and mitigating pandemic-level harms. Pandemic-level harms have the potential to spread uncontrollably and endanger the health and lives of populations around the world. Emerging international guidance and national policy focus on overseeing wet-lab capabilities that enable or enhance pandemic-relevant properties, such as transmissibility, virulence, or immune escape, in pathogens (National Research Council, 2004; World Health Organization, 2022; Bulletin of the Atomic Scientists, 2024; National Institutes of Health, 2024; The White House OSTP, 2024, 2025).

Such frameworks for wet-lab oversight of research that could be reasonably anticipated to create novel pandemic-level harms focus on interventions early in the research lifecycle and have pivoted from expert *post hoc* assessments to upstream risk–benefit reviews (The White House OSTP, 2024; National Academies of Sciences, Engineering, and Medicine, 2025). For wet-lab dual-use research, upstream risk–benefit review is well-established. For instance, institutional oversight bodies, such as Biosafety Officers on Institutional Biosafety Committees, and federal funding agencies, such as the National Institutes of Health (NIH) or the Centers for Disease Control and Prevention (CDC), review proposals before any work is carried out in the laboratory (National Institutes of Health, 2024).

### The need to move biological AI model risk reduction upstream

With current risk mitigation for BAIMs focused on the post-development stage, and dual-use frameworks highlighting the need for earlier assessments, it is crucial to move toward a regime in which BAIM risk review occurs upstream in the lifecycle before a model is developed.

We argue that pre-development risk–benefit review (RBR) for a subset of BAIMs is a crucial missing component to reducing real-world risks and that this is feasible to implement widely. Such reviews should occur for both academic and commercial model development. The funding stage poses a well-suited point of control for this.

Many characteristics render pre-development RBR particularly promising for BAIMs compared to general-purpose AI (GPAI). For GPAI, evaluations and built-in technical safeguards have been established as the main avenue to reducing risks, and risk management approaches for BAIMs may be unduly modelled after this.

Importantly, many BAIMs are purpose-built for specific capabilities, and these capabilities are known at model conception. For instance, the model EVEScape was purpose-built to predict viral mutations that can evade host immune responses (Thadani et al., 2023). In contrast, GPAI capabilities like bioweapon ideation emerge unintentionally. These purpose-built characteristics of BAIMs enable estimating the dual-use potential of a BAIM at the conception stage.

Since BAIMs exhibit substantial heterogeneity in their dual-use capabilities and underlying model architectures, it is difficult to develop broadly applicable model safeguards and evaluations. Some of these would additionally require different forms of laboratory validation with safe proxy organisms (Ikonomova et al., 2026). Also, establishing evaluations and safeguards is resource-intensive, and thus less feasible for many BAIM developers, who tend to be academics or at other research institutions (Atanasov et al., 2026).

Upstream RBRs can support responsible model developers by setting clear expectations on risk management practices from the beginning. It can steer new BAIM development toward directions that pose fewer dual-use concerns, allow factoring in resources for subsequent evaluations or safeguards, and call for responsible model dissemination practices such as managed access (Carter and Butchello, 2026). It could require developers to implement less resource-intensive safeguards before a model is trained, most prominently, training “data filtering” (Brixi et al., 2025; Hayes et al., 2025; Atanasov et al., 2026). We believe a governance regime in which RBRs for BAIMs ensure that security is integrated during model inception would be more fair to model developers than imposing strong security and risk-reduction requirements after development, for instance, at the publication stage.

There is a need for standardized and consistent RBR practices. Currently, where RBRs did occur, responsible developers had to make *ad hoc* decisions on misuse risks and mitigation. Existing wet-lab frameworks are inappropriate for overseeing BAIMs, given inherent differences in risk and risk reduction, underscoring the importance of developing standardized, readily applicable pre-development RBR frameworks for BAIMs.

### Existing discussions of upstream risk–benefit review

Few policies have addressed upstream RBRs for BAIMs to date. The 2024 DURC/PEPP (Dual-Use Research of Concern / Pathogens with Enhanced Pandemic Potential) policy recommended voluntary institutional RBR, and accompanying risk mitigation plans for “*In Silico* Models and Computational Approaches […] directly enabling the design of a PEPP or a novel biological agent or toxin” (The White House OSTP, 2024). In May 2025, Executive Order (EO) 14,292 directed that the 2024 DURC/PEPP policy be revised or replaced (The White House OSTP, 2025). At the time of writing, the revised or replaced policy had not been released.

A 2025 statement from UK Research and Innovation (UKRI) similarly points out “computational, and predictive technologies in pathogen or toxin-relevant research, such as artificial intelligence (AI)” as research with potential misuse risks (UKRI, 2025). It recommends self-governance, encouraging researchers and their institutions “to seek independent expert advice and independent oversight” to ensure “anticipated benefits significantly outweigh the risks.” Still, it mentions that UKRI “funding terms and conditions” ensure consideration of misuse risk and include provisions on appropriate risk management, but no further information on the UKRI implementation is available.

Outside of government policies, some notable stakeholders have highlighted the importance of upstream RBRs. A National Academies workshop focused on dissemination practices for *in silico* biological research noted that “participants emphasized the importance of addressing steps earlier in the research lifecycle, such as upstream oversight, risk assessment, and development of responsible research norms” and the “importance of comprehensive biosecurity reviews […] from early engagement by funders, institutions, and researchers.” It highlighted the need for a modular risk–benefit assessment framework appropriate for the full spectrum of BAIM developers (National Academies of Sciences, Engineering, and Medicine, 2025).

The first key recommendation from a report by the RAND Corporation (RAND) and the Centre for Long-Term Resilience that developed a detailed risk-scoring index for BAIMs was that “Developers and funders should use the Global Risk Index rubrics to assess tools for misuse-relevant capabilities before funding and developing tools” (Webster et al., 2025). The rubrics were developed for post-development risk-assessment, so their applicability to the pre-development stage is yet to be explored.

For funders, the Nuclear Threat Initiative (NTI) runs the International Bio Funders Compact, where several organizations that fund AI-related projects committed to conducting pre-funding biosecurity and biosafety reviews (Sengupta et al., 2024). Recent NTI implementation guidance for assessing biosecurity and biosafety risks pre-funding was not yet applicable to *in silico* work (Yassif et al., 2025).

Calls for upstream RBRs have also increased among research communities. Members of the Responsible AI x Biodesign initiative have recommended coordinating an “interdisciplinary effort to develop pre- and post-development review processes to identify and evaluate capabilities of concern in relevant tools and explore developing and recommending proportional risk mitigation measures” (Rivera et al., 2025).

While literature and policy frameworks increasingly highlight the importance of upstream RBRs, public resources detailing what such reviews would specifically cover remain scarce.

## Framework for upstream risk–benefit review

### Overview of the five risk–benefit review components

**Figure 1:**
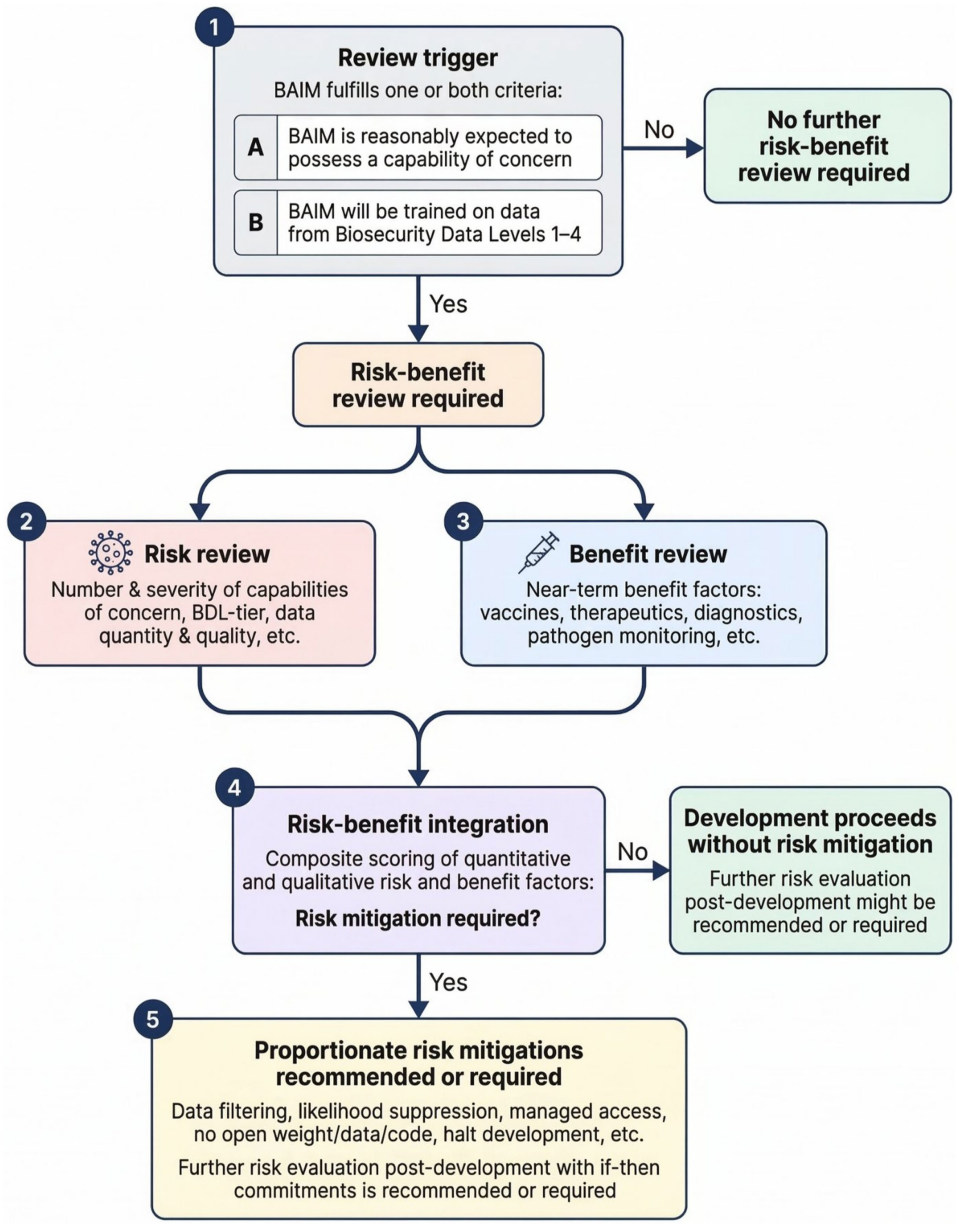
Upstream risk–benefit review (RBR) framework for biological AI models (BAIMs). A BAIM enters an RBR process if it meets one or both trigger criteria: **(A)** It is reasonably expected to possess a capability of concern, or **(B)** It will be trained on data from Biosecurity Data Levels 1–4. If triggered, the model undergoes parallel risk and benefit reviews (steps 2 and 3), who are subsequently integrated into a composite scoring (step 4). If this identifies substantial risks, proportionate mitigations are recommended or required (step 5). The framework can be applied across academic institutions, commercial developers, and federal, philanthropic and private funding bodies.

We propose that an RBR process should consist of the following five components (Figure 1):

a) Review trigger
b) Risk review
c) Benefit review
d) Risk and benefit integration
e) Proportionate mitigations recommended

We define criteria that should trigger an RBR, followed by key characteristics to consider in the respective risk and benefit reviews, integration of those reviews, and discuss proportionate risk mitigation measures that can be recommended. While this framework outlines key characteristics of the process, it does not pose a quantifiable risk–benefit scoring system. It can be applicable across academic, commercial, and other forms of model development.

### Risk–benefit review trigger

The criteria that trigger a model to undergo upstream RBR should aim to capture every model that would end up posing substantial misuse-risk, while not being overly broad and including a large number of models that will turn out not to pose any risk. The latter is important, as the majority of BAIMs developed to date pose no substantial risks (Webster et al., 2025), and their development should not risk being delayed by RBR processes. As BAIM development is considered beneficial by default, the trigger criteria are based on surfacing potential misuse risk.

We propose two buckets of criteria that trigger an RBR if one or both are fulfilled (further defined below):

1. The model can be reasonably expected to possess a capability of concern.
2. The model will be trained on sensitive biological data.

Firstly, it should be considered whether a model can be reasonably expected to possess a capability of concern. We can draw on existing literature spelling out capabilities of concern for biological AI models. Pannu et al. identified several “capabilities of concern” that were adapted from the experimental outcomes of concern of the 2024 DURC/PEPP policy (Pannu et al., 2025a). These focus on capabilities that enhance the pandemic potential of pathogens, for instance, by increasing transmissibility or virulence. An expert ranking of the most concerning capabilities highlighted the prediction of immune escape and host range, among others (Pannu et al., 2024, 2025b). Similarly, Webster et al. introduce a detailed taxonomy of capabilities of concern of biological AI models, based on which category a particular model falls into (e.g., protein engineering, pathogen property prediction, etc.) (Webster et al., 2025). Another RAND report highlights five viral capabilities with particular AI-modification risks: “Altered host range or tropism, increased genome replication, immune or medical countermeasure evasion, increased environmental stability, and increased transmission dynamics” (Williams et al., 2026). Given the severity of societal harm posed by novel pandemic harms, capabilities of concern have focused on capabilities that could increase risks from pandemic pathogens. Another type of capability of concern a model can possess is enabling the circumvention of existing biosecurity measures. For instance, creating functional protein homologs with differing sequences could undermine nucleic acid synthesis screening (Wittmann et al., 2025; Ikonomova et al., 2026).

While it cannot be known definitively whether a model will possess a capability until it is developed and evaluated, many biological AI models are developed with the goal of expressing a particular capability. Model developers should expect to succeed in developing the intended capability insofar as this can be reasonably anticipated. This is analogous to how the 2024 DURC/PEPP policies asked researchers to reasonably anticipate whether their experiment could lead to an outcome of concern, before the experiment was actually attempted (The White House OSTP, 2024).

Also, some biological AI models are not developed to express a narrow capability. Instead, they are foundation models with broadly applicable capabilities. Capabilities of concern of such models would not be as apparent as for narrow models, and researchers should carefully assess whether an identified capability of concern applies.

Secondly, it should be considered whether a model will be trained on sensitive biological data. The capability of an AI model to perform a specific task is dependent on domain-related training data, and models become incrementally worse at generalizing beyond their training data (Benegas et al., 2025; Tang et al., 2025). For instance, excluding viral sequences reduces a model’s capability to perform virus-related tasks like mutational fitness prediction (Brixi et al., 2025; Hayes et al., 2025). In turn, including or fine-tuning on pathogen-related data improves performance on respective misuse-relevant tasks (Dip et al., 2024; Black et al., 2025; Wei et al., 2025; Zhang et al., 2025b). In a 2025 Asilomar entreaty, more than 100 renowned scientists called for a system of data access controls to limit harmful AI-biology capabilities (Bromberg et al., 2025). This idea was subsequently developed into Biosecurity Data Levels (BDLs), a five-tier framework (BDL 0–4) that categorizes datasets by their potential to enable misuse-relevant AI capabilities and links progressively stricter access control requirements to each tier (Berke, 2026; Bloomfield et al., 2026a,b).

If any data falling into BDL 1–4 is planned to be included in a model’s training data, this should trigger an upstream RBR for the model developers. While data provide a proxy measure of expected model capabilities, they require less subjective judgment from model developers than reasonably anticipated model capabilities.

How exactly an RBR should be triggered by these criteria depends on where the model will be developed. In academic settings, analogous to how this works for wet-lab research, the principal investigator should flag the need for review and would then be supported by an institutional review board. The review should also be triggered by federal and private funding bodies when identified during grant review. In commercial settings, a company should have an ethics board and guidelines that set review requirements and support developers throughout the process. While this process should be voluntary to begin, if its value, effectiveness, and feasibility are demonstrated, RBR for BAIMs that could pose pandemic-level harms should become a requirement as a means of protecting the public from high-consequence biological events.

### Risk review characteristics

Once the need for an RBR is triggered, a structured process should assess the expected severity of the risks posed by that BAIM. It is important to systematize and standardize the language and criteria that reviewers use for this. We outline general criteria to consider rather than providing a quantitative rubric for risk scoring.

Several dimensions related to capabilities of concern should be considered. A greater number of capabilities of concern that the BAIM is reasonably anticipated to possess should result in a higher risk score. Also, the severity of a particular capability of concern at hand is crucial. Pannu et al. group their capabilities of concern into Category 1 and Category 2, modelled after the DURC/PEPP policy (Pannu et al., 2025a). Category 2 capabilities revolve around enhancing pathogen characteristics such as transmissibility for human-infecting viruses with pandemic potential, in particular. Similarly, Webster et al. score capability of concern risk levels from “very low” to “critical,” in part dependent on what pathogen properties they alter (Webster et al., 2025). Additionally, the generalizability of a capability across a range of biological agents should be considered. Meaning, the more a model is expected to generalize to a wide range of pathogens, particularly those with pandemic potential, the higher the risk score should be, as this opens up a larger range of threat pathways. Lastly, the likelihood that a model will express one or multiple of the respective capabilities of concern should be considered. While the trigger for an RBR only requires that it is reasonably expected the model will possess a capability of concern, the review should quantify how likely that is, with higher probabilities resulting in a higher risk score. Also, particularly for biological foundation models, developers should assess whether a model could be easily modified to express a capability of concern, for instance, by fine-tuning on sensitive pathogen data.

For sensitive pathogen data, the higher the BDL for the data included in a BAIMs training data, the higher the risk score. The amount and quality of sensitive pathogen data should also be considered, with larger quantities and higher quality data resulting in higher risk scores (Berke et al., 2025). Additionally, it should be considered whether the training data is openly available or exclusively available to model developers. If sensitive data is exclusively available to model developers, a different model cannot easily be retrained or fine-tuned on that data. This can increase the risk score, as it is less likely that the resulting model capability would already be present in an existing model trained on open data. In addition, there is significant risk from fine-tuning a model on openly available sensitive data if model weights are openly available (Righetti, 2025). Lastly, there is some remaining risk that even if weights are closed, when sensitive training data and model code are openly available, the model can be retrained, for instance, with support from AI agents.

It is important to consider whether a BAIM under review is likely to exceed the capabilities of concern of existing and openly available BAIMs. While this can be hard to assess before model development, researchers should assume they will meet their development goals. Close risk review is warranted if it is plausible that the model will exceed the state of the art.

The risk score should also vary based on the accessibility developers intend for their model. The higher the level of openness (ranging across open weights, open training code, open data, open inference code, web interface, API (Application Programming Interface) access, or tiered managed access (Atanasov et al., 2026)), the higher the risk score should be, as it becomes easier for malicious actors to use and modify the model undetected. The risk score should also be reduced if developers intend to implement safeguards for their model.

### Benefit review characteristics

Assessing benefits using clear operational criteria remains underdeveloped in life science research, and there are no existing concepts for BAIMs. Related wet-lab frameworks focus on risk management and assume minimizing risk translates to maximizing benefits (Yassif et al., 2025). Still, a 2025 RAND report proposed a structured tool based on benefit factors and concrete indicators that reviewers could score (Williams et al., 2025). This included expected improvements for existing medical countermeasures, enabling novel therapeutics or vaccine targets, contributions to diagnostics, early-warning or predictive modeling, and pathogen monitoring, among others. For each of these benefit factors, it can be considered how the research affects expected products-to-market, time-to-impact, technology readiness levels, and the marginal improvement over existing methods.

When transferring this approach to BAIMs, a benefit-review should score which of these benefit factors would be affected by a particular proposed BAIM and to what extent. Akin to how this has been discussed for wet-lab DURC, developers should demonstrate a clear near-term public health application for their proposed BAIM, specifying the primary benefit pathway, the timeframe, and the plausibility conditions for realizing it (Lipsitch and Galvani, 2014). The benefit scoring should also weigh whether comparable benefits could be achieved through approaches that do not require similar capabilities of concern (Lipsitch, 2014). Some benefit-review findings and scores will translate across certain BAIM categories, reducing the burden of in-depth benefit reviews for an individual model.

### Risk and benefit integration

Risk- and benefit-scores should be integrated into a composite score that constitutes the bottom-line assessment. In addition to a quantitative component, this should integrate crucial qualitative considerations from the risk and benefit reviews. When this concludes that a model under development poses relevant risks, it is crucial that concrete and proportionate risk mitigation measures are recommended and subsequently implemented during the development and release process. This is a crucial step, aiming to tip the balance for a BAIM from the risk-dominant to the benefit-dominant side. Dependent on a BAIMs risk profile, different risk mitigations might be particularly warranted or effective.

### Potential risk mitigations

While risk mitigations are a nascent field for BAIMs, we can draw on literature developed for frontier GPAI and DURC to provide an overview of options (Frontier Model Forum, 2025).

First, some methods limit the actual capabilities of concern a model possesses. An established technique is “data filtering,” where data that enables capabilities of concern is omitted from a model’s training data. For instance, the models ESM3-open, Evo 1, and Evo 2 omitted some viral sequencing data, leading to reduced performance on misuse-related tasks while preserving overall model function (Nguyen et al., 2024; Brixi et al., 2025; Hayes et al., 2025). Upstream RBRs are particularly relevant for recommending data filtering, as it has to be implemented before model training. The exclusion of certain data is not resource-intensive and could be easily identified when an RBR surfaces data falling into BDL 1–4 is planned to be included in model training. Still, capabilities of concern can be regained by reintroducing omitted training data or via jailbreaking techniques (Black et al., 2025; Wei et al., 2025; Zhang et al., 2025b).

Another safeguard applied at the training-stage is “likelihood suppression,” which penalizes the learning of harmful sequences (Wang et al., 2026). Like data filtering, this also needs to be accounted for before model training and can be surfaced by an upstream RBR. Other methods to limit capabilities of concern, like “unlearning,” can be applied post-training but are less explored and likely less robust to circumvention (Li et al., 2024; Łucki et al., 2025; Dettman et al., 2026).

Second, there are methods where a model possesses capabilities of concern, but these are prevented from being expressed. For instance, the AlphaFold Server explored “screening and filtering processes” and “blocking a small number of viral protein sequences” (Griffin et al., 2024). This can occur by screening model inputs, but also by detecting outputs of generated sequences too close to known harmful ones (Wang et al., 2026). All these safeguards can only be meaningfully applied to non-openly available models, as they must be embedded in a controlled environment such as an API. For open-weight models, safeguards can easily be circumvented (Dong et al., 2025).

Third, some methods are not related to modulating model capabilities but mitigate risks by controlling the model’s availability. In particular, “managed access” regimes limit model access to validated, legitimate users, thus preventing unfettered access to capabilities of concern. Providing access to a model via a controlled environment, such as an API interface, also enables monitoring of usage patterns and ensures that built-in technical guardrails remain intact. Alternatively, since API hosting and managed access platforms can be resource-intensive, a managed access approach for making source code or training data available can also be considered. Managed access can include a tiered system in which increasingly stringent identity and legitimacy verification, and increasingly secure computational environments, are required as the model’s misuse potential increases (Carter and Butchello, 2026). Still, managed access settings are vulnerable to data and code being illicitly copied and distributed. Trusted research environments (TREs) are virtual platforms where researchers run code in a closed environment containing sensitive data or BAIMs without needing to directly access or export data or code. Custodians then review aggregate outputs before extraction from the TRE. This reduces the risk of data or BAIMs leaking in managed access settings. For high risk BAIMs and data, TREs pose a promising mitigation measure, with a strong existing precedent in the health data domain (Bloomfield et al., 2026b). Upstream RBRs could recommend that a BAIM should be made available through a managed access regime or via a TRE.

A less resource-intensive measure that does not monitor model access is not to openly release model weights, data, or training/inference code, so that subsequent modifications that increase misuse potential are harder to perform. Another available risk mitigation measure is to omit methodological details or information from the publication that directly contain misuse-relevant details (see Wittmann et al., 2025, for an example) or could serve as a blueprint for high-misuse work. Models could also be developed and made available only to trusted stakeholders. As a last resort, upstream RBR can recommend that a model should not be developed at all if it would be highly misuse-enabling, with few benefits, and no risk mitigation measures would be adequate.

While some risk mitigations can only be implemented downstream of model training, upstream RBRs allow model developers to consider the most efficient measures to ensure security early and to factor in sufficient resources for later implementation. The need for implementing post-training risk mitigation can also be made conditional on evaluation results. An upstream RBR could establish clear “if-then” commitments for developers, tying evaluation results to certain post-training risk mitigations (Karnofsky, 2024). For instance, a developer could pre-commit to that if evaluations show a model can predict viable high-fitness variants for viruses outside of the training data, the model will only be made available via a managed access platform.

It remains important to conduct rigorous efficacy studies for these various risk mitigation measures to assess their contribution to real-world risk reduction.

## Discussion

### Toward future risk–benefit review implementation

We outline an initial framework for RBR for BAIMs that highlights key considerations and actions that are qualitative in nature. More research and data are required to develop quantitative scores and decision thresholds for reviewers. To ensure future operationalization, more work from experts is needed, including authoritative working groups that define capabilities of concern, define BDLs in more detail, determine quantifiable risk, and benefit-review criteria, and set best practices for risk mitigation. Semi-quantifiable criteria could also be developed as a first step. Experts have developed a proposed version centered around AI-enabled modification of select viral capabilities (Williams et al., 2026). Subsequently, RBR operational feasibility and value for risk reduction should be demonstrated via validation case studies. This can also be supported by creating hypothetical case studies or using historical examples of BAIM development to simulate how an RBR would have altered risk management outcomes, for instance, as included in Williams et al., 2025, for laboratory DURC/PEPP research.

We recommend that RBR frameworks should be refined and implemented internationally across BAIM developers and funders, including via federal, philanthropic, or private funders that establish respective requirements.

At the national level, this would involve establishing RBR recommendations for federally funded BAIM development, which could evolve into mandatory requirements once processes are established. In the US, this could be advanced through NIH’s “Initiative to Modernize and Strengthen Biosafety Oversight” (National Institutes of Health, 2025) or measures arising from the Executive Order on “Improving the Safety and Security of Biological Research” (The White House OSTP, 2025). Similarly, Institutional Biosafety Committees and Biosafety Officers should be supported through training materials in building expertise in assessing *in silico* research. In the UK, UKRI could provide more in-depth guidance on how the risk-review mechanisms for researchers, as well as for the Biotechnology and Biological Sciences Research Council (BBSRC) and Medical Research Council (MRC), will be implemented (UKRI, 2025). Other nations should explore how their national dual-use life sciences oversight could be adapted to cover *in silico* approaches. Internationally, the World Health Organization (WHO) should start incorporating recommendations on *in silico* research in its global guidance framework on dual-use research (World Health Organization, 2022).

In parallel, we recommend that private and philanthropic funders, as well as commercial model developers, should build out their RBR processes. This could include expanding the NTI Bio Funders Compact to include *in silico* approaches, as well as expanding such review commitments to a larger number of funders (Sengupta et al., 2024). Major biological AI model developers, such as the Chan Zuckerberg Biohub or the Arc Institute, should test and adapt similar review processes. It is important that review processes are vetted through real-world applications to ensure feasibility, clarity, and reliability. An openly available “BAIM quick scan tool” that incorporates risk–benefit scoring metrics could support model developers with a first-pass RBR, akin to what exists for wet lab dual-use and biosafety assessment (Dutch Biosecurity Office, 2021; World Health Organization, 2024).

Given the rapid pace of progress in BAIM development, frameworks should be modular and adaptable to avoid being quickly out of date. These efforts could be accompanied by an anonymized registry of decisions that highlights how peers handled similar cases, and how decisions were made for RBRs and necessary risk mitigations. This would reduce duplicate work and identify patterns for iterative revisions.

Lastly, in the absence of existing frameworks for upstream RBR, even provisional processes focused on identifying and reducing risks from the highest-risk models could provide substantial risk reduction relative to the status quo.

### Limitations of risk–benefit reviews

Many points of criticism that apply to wet-lab DURC oversight also extend to *in silico* aspects. For instance, terms like “reasonably anticipated” can be subjective and applied differently even among experts (Greene et al., 2025). This is exacerbated for *in silico* approaches, where the ultimate capabilities of a model are not known before development and evaluation and can only be approximated, leading to increased uncertainty. Ensuring consistency across reviewers is thus a central challenge. Consistency can be supported through standardized criteria with anchoring examples, reviewer training materials, and cases being reviewed by at least two independent reviewers. The anonymized registry of review decisions proposed above would enable empirical assessment of inter-rater reliability and allow criteria to be recalibrated where variance is high.

While reviews should extend beyond qualitative components, accurately quantifying risks and benefits that might only arise years down the line is challenging. Additionally, RBRs would require incorporating knowledge on AI, Biosecurity, and national security, a combination that is very scarce and hard to scale across review bodies. Classified intelligence relevant to RBRs is often unavailable to stakeholders.

Additionally, conflicts of interest can skew the incentives of review bodies to avoid conducting reviews or overemphasize the benefits. Reviews at private entities might prioritize commercial interest over delaying model development or diverting resources to risk review and mitigation. For academic developers, principal investigators would be initiating RBRs for their models. They could forego flagging models if this is expected to cost additional resources or jeopardize future funding. Funders control a natural leverage point but may lack in-house review expertise. National security stakeholders hold classified insights but have limited channels to feed them into academic or commercial RBR. Aligning these incentives and providing structured information sharing are central to making RBR work in practice.

RBRs risk being too complicated to implement or not being attuned to the realities model developers face. This might reduce adoption or make them impractical to apply. Vetting processes and best practice sharing are needed to reduce such concerns.

Lastly, overly broad RBR triggers risks capturing an overly wide range of models and slowing down the development of BAIMs without meaningful dual-use risks. Given their potential for accelerating science and medicine, it is important that BAIM development can continue rapidly. Thus, it is crucial that RBR triggers are calibrated carefully and highly selectively, and that review processes occur swiftly. Mandatory, but slow or inconsistent RBR might also displace BAIM development toward jurisdictions or institutions without or with poor oversight, potentially increasing overall risk. Requiring BAIM RBR in addition to the existing review obligation for Institutional Review Boards, without providing sufficient capacity, could additionally degrade the quality of both.

## Conclusion and call to action

We believe that governance approaches for BAIMs must extend beyond post-development approaches like evaluations and safeguards to upstream RBRs. We recommend that such a framework should incorporate criteria for an RBR trigger, qualitative and quantitative metrics for the risk and benefit review arms, as well as their integration into a composite assessment, and recommend proportionate risk mitigation measures. RBR should be triggered when a model is reasonably anticipated to have capabilities of concern or include dual-use pathogen (BDL 1–4) training data. Given the large-scale societal consequences they could cause, capabilities of concern should be centered around enhancing pathogens with pandemic potential or reducing human immunity to them.

We believe that RBRs would only trigger for a small share of BAIMs and would support responsible developers by setting clear expectations from the outset, rather than imposing burdensome requirements after a model has been developed. Still, BAIM RBRs are a nascent concept, and further developing and operationalizing them requires substantial expert input. Authoritative working groups should clarify capabilities of concern, establish quantitative and qualitative criteria for risk and benefit review, and validate the efficacy of recommended risk mitigations. Such an effort could be coordinated by a novel entity akin to the Frontier Model Forum (Frontier Model Forum, 2023), but for BAIMs instead of frontier GPAI, and should be coordinated with the International Network for Advanced AI Measurement, Evaluation, and Science (Center for AI Standards and Innovation, 2026).

We call on academic and commercial model developers and their governmental, philanthropic, and private funders to develop, pilot, and iterate through upstream RBR processes to ensure adequate future risk management.

## Data Availability

The original contributions presented in the study are included in the article/supplementary material, further inquiries can be directed to the corresponding authors.

## References

[ref1] Atanasov D. Zanichelli N. Denain J.-S. (2026). Expanding our analysis of biological AI models. Available at: https://epoch.ai/blog/expanding-our-analysis-of-biological-ai-models (Accessed March 13, 2026).

[ref2] Baker D. Church G. (2024). Protein design meets biosecurity. Science 383, 349–349. doi: 10.1126/science.ado1671 38271530

[ref3] Benegas G. Ye C. Albors C. Li J. C. Song Y. S. (2025). Genomic language models: opportunities and challenges. Trends Genet. 41, 286–302. doi: 10.1016/j.tig.2024.11.013 39753409

[ref4] Bennett N. R. Watson J. L. Ragotte R. J. Borst A. J. See D. L. Weidle C. . (2025). Atomically accurate de novo design of antibodies with RFdiffusion. Nature 649, 183–193. doi: 10.1038/s41586-025-09721-5, 41193805 PMC12727541

[ref5] Berke A. (2026). Risk-Based Categorization and Governance of Biological Data in AI Systems. Baltimore: Johns Hopkins Center for Health Security.

[ref6] Berke A. Crawford F. W. Webster T. Smith J. Zakaria S. Nevo S. (2025). Data and AI-enabled biological design: risks related to biological training data and opportunities for governance. Available online at: https://www.rand.org/pubs/perspectives/PEA3886-1.html (Accessed March 13, 2026).

[ref7] Biswas S. Khimulya G. Alley E. C. Esvelt K. M. Church G. M. (2021). Low-N protein engineering with data-efficient deep learning. Nat. Methods 18, 389–396. doi: 10.1038/s41592-021-01100-y, 33828272

[ref8] Black J. R. M. Hanke M. S. Maiwald A. Hernandez-Boussard T. Crook O. M. Pannu J. (2025). Open-weight genome language model safeguards: assessing robustness via adversarial fine-tuning. doi: 10.48550/arXiv.2511.19299

[ref9] Bloomfield D. Berke A. Hanke M. S. Maiwald A. Black J. R. M. Webster T. . (2026a). Securing dual-use pathogen data of. Concern. doi: 10.48550/arXiv.2602.08061

[ref10] Bloomfield D. Black J. R. M. Crook O. Brandes N. Hanke M. S. Inglesby T. V. . (2026b). Biological data governance in an age of AI. Science 391, 558–561. doi: 10.1126/science.aeb2689, 41643006

[ref11] Brixi G. Durrant M. G. Ku J. Poli M. Brockman G. Chang D. . (2025). Genome modeling and design across all domains of life with Evo 2:638918. doi: 10.1101/2025.02.18.638918 PMC13128491 41781614

[ref12] Bromberg Y. Altman R. Imperiale M. Horvitz E. Dus M. Townshend R. . (2025). 3.1 artificial intelligence and the future of biotechnology. Available online at: https://hdl.handle.net/1911/118555 (Accessed November 18, 2025).

[ref13] Bulletin of the Atomic Scientists (2024). A Framework for Tomorrow’s Pathogen Research. Available online at: https://thebulletin.org/pathogens-project/ (Accessed March 13, 2026).

[ref14] Carter S. R. Butchello G. (2026). A Framework for Managed Access to Biological AI Tools. Washington: The Nuclear Threat Initiative.

[ref15] Center for AI Standards and Innovation (2026). International Network for Advanced AI Measurement, Evaluation, and Science Publishes Consensus Areas on Practices for Automated Evaluations. Gaithersburg: NIST.

[ref16] Dettman J. Lathrop E. Attal-Juncqua A. Nicotra M. Berke A. (2026). Prioritizing feasible and impactful actions to enable secure AI development and use in biology. Biotechnol. Bioeng. doi: 10.1002/bit.70132, 41546597

[ref17] Dip S. A. Shuvo U. A. Chau T. Song H. Choi P. Wang X. . (2024). Patholm: identifying pathogenicity from the DNA sequence through the genome foundation model. doi: 10.48550/arXiv.2406.13133

[ref18] Dong Y. Mu R. Zhang Y. Sun S. Zhang T. Wu C. . (2025). Safeguarding large language models: a survey. Artif. Intell. Rev. 58:382. doi: 10.1007/s10462-025-11389-2, 41114380 PMC12532640

[ref19] Dutch Biosecurity Office (2021). Dual-use Quickscan. Available online at: https://dualusequickscan.com/en/ (Accessed March 13, 2026).

[ref20] Fan J. Zhou Z. Jin R. Cong L. Wang M. Zhang Z. (2025). Safeprotein: red-teaming framework and benchmark for protein foundation models. doi: 10.48550/arXiv.2509.03487

[ref21] Frontier Model Forum (2023). Frontier Model Forum. Washington: Frontier Model Forum.

[ref22] Frontier Model Forum (2025). Frontier Mitigations. Washington: Frontier Model Forum.

[ref23] Gonzalez-Isunza G. Jawaid M. Z. Liu P. Cox D. L. Vazquez M. Arsuaga J. (2023). Using machine learning to detect coronaviruses potentially infectious to humans. Sci. Rep. 13:9319. doi: 10.1038/s41598-023-35861-7, 37291260 PMC10248971

[ref24] Greene D. Alexanian T. Palmer M. J. (2025). Mapping variation in dual use risk assessments of synthetic biology projects. Front. Bioeng. Biotechnol. 13:1620678. doi: 10.3389/fbioe.2025.1620678, 40895722 PMC12392780

[ref25] Griffin C. Howard H. Paterson A. Swanson N. Bloxwich D. Jumper J. . (2024). Our approach to biosecurity for AlphaFold 3. Available online at: https://storage.googleapis.com/deepmind-media/DeepMind.com/Blog/alphafold-3-predicts-the-structure-and-interactions-of-all-lifes-molecules/Our-approach-to-biosecurity-for-AlphaFold-3-08052024

[ref26] Gurev S. Youssef N. Jain N. Marks D. S. (2025). Variant effect prediction with reliability estimation across priority viruses. 2025.08.04.668549. doi: 10.1101/2025.08.04.668549

[ref27] Hayes T. Rao R. Akin H. Sofroniew N. J. Oktay D. Lin Z. . (2025). Simulating 500 million years of evolution with a language model. Science 387, 850–858. doi: 10.1126/science.ads0018, 39818825

[ref28] Hie B. Zhong E. D. Berger B. Bryson B. (2021). Learning the language of viral evolution and escape. Science 371, 284–288. doi: 10.1126/science.abd7331, 33446556

[ref29] Ikonomova S. P. Wittmann B. J. Piorino F. Ross D. J. Schaffter S. W. Vasilyeva O. . (2026). Experimental evaluation of AI-driven protein design risks using safe biological proxies. 2025.05.15.654077. doi: 10.1101/2025.05.15.654077

[ref30] Ingraham J. B. Baranov M. Costello Z. Barber K. W. Wang W. Ismail A. . (2023). Illuminating protein space with a programmable generative model. Nature 623, 1070–1078. doi: 10.1038/s41586-023-06728-8, 37968394 PMC10686827

[ref31] Johnson C. Y. (2025). Inside the debate over a tech breakthrough raising questions about life itself. Wash. Post. Available online at: https://www.washingtonpost.com/science/2025/11/11/ai-designed-viruses-bacteria-life/ (Accessed March 13, 2026).

[ref32] Karnofsky H. (2024). If-then commitments for AI risk reduction. Carnegie Endowment for International Peace. Available online at: https://carnegieendowment.org/research/2024/09/if-then-commitments-for-ai-risk-reduction (Accessed March 13, 2026).

[ref33] King S. H. Driscoll C. L. Li D. B. Guo D. Merchant A. T. Brixi G. . (2025). Generative design of novel bacteriophages with genome language models. doi: 10.1101/2025.09.12.675911 42561074

[ref34] Li N. Pan A. Gopal A. Yue S. Berrios D. Gatti A. . (2024). The WMDP benchmark: measuring and reducing malicious use with unlearning. doi: 10.48550/arXiv.2403.03218

[ref35] Lipsitch M. (2014). Can limited scientific value of potential pandemic pathogen experiments justify the risks? mBio 5:10-1128. doi: 10.1128/mbio.02008-14 PMC4205796 25316701

[ref36] Lipsitch M. Galvani A. P. (2014). Ethical alternatives to experiments with novel potential pandemic pathogens. PLoS Med. 11:e1001646. doi: 10.1371/journal.pmed.1001646, 24844931 PMC4028196

[ref37] Łucki J. Wei B. Huang Y. Henderson P. Tramèr F. Rando J. (2025). An adversarial perspective on machine unlearning for AI safety. doi: 10.48550/arXiv.2409.18025

[ref38] Merchant A. T. King S. H. Nguyen E. Hie B. L. (2026). Semantic design of functional de novo genes from a genomic language model. Nature 649, 749–758. doi: 10.1038/s41586-025-09749-7, 41261132 PMC12804078

[ref39] National Academies of Sciences, Engineering, and Medicine (2025). Disseminating In Silico and Computational Biological Research: Navigating Benefits and Risks: Proceedings of a Workshop. Washington: National Academies Press.

[ref40] National Institutes of Health (2024). NIH guidelines for research involving recombinant or synthetic nucleic acid molecules (NIH guidelines). Available online at: https://osp.od.nih.gov/wp-content/uploads/NIH_Guidelines.htm (Accessed March 13, 2026).

[ref41] National Institutes of Health (2025). NIH launches initiative to modernize and strengthen biosafety oversight | National Institutes of Health (NIH). Available online at: https://www.nih.gov/about-nih/nih-director/statements/nih-launches-initiative-modernize-strengthen-biosafety-oversight (Accessed April 20, 2026).

[ref42] National Research Council (2004). Biotechnology Research in an Age of Terrorism. Washington: National Academies Press. 25057686

[ref43] Nguyen E. Poli M. Durrant M. G. Kang B. Katrekar D. Li D. B. . (2024). Sequence modeling and design from molecular to genome scale with Evo. Science 386:eado9336. doi: 10.1126/science.ado9336, 39541441 PMC12057570

[ref44] Pannu J. Bloomfield D. MacKnight R. Hanke M. S. Zhu A. Gomes G. . (2025a). Dual-use capabilities of concern of biological AI models. PLoS Comput. Biol. 21:e1012975. doi: 10.1371/journal.pcbi.1012975, 40338934 PMC12061118

[ref45] Pannu J. Gebauer S. L. Bradley H. A. Woods D. Bloomfield D. Berke A. . (2025b). Defining hazardous capabilities of biological AI models: expert convening to inform future risk assessment. Available at: https://www.rand.org/pubs/conf_proceedings/CFA3649-1.html (Accessed September 23, 2025).

[ref46] Pannu J. Gebauer S. McKelvey G. Jr. Cicero A. Inglesby T. (2024). AI could pose pandemic-scale biosecurity risks. Here’s how to make it safer. Nature 635, 808–811. doi: 10.1038/d41586-024-03815-2, 39572723

[ref47] Rapp J. T. Bremer B. J. Romero P. A. (2024). Self-driving laboratories to autonomously navigate the protein fitness landscape. Nat. Chem. Eng. 1, 97–107. doi: 10.1038/s44286-023-00002-4, 38468718 PMC10926838

[ref48] Responsible AI x Biodesign (2024). Community values, guiding principles, and commitments for the responsible development of AI for protein design. Available at: https://responsiblebiodesign.ai/ (Accessed March 13, 2026).

[ref49] Righetti L. (2025). Dual-use AI capabilities and the risk of bioterrorism. Available at: https://www.governance.ai/research-paper/dual-use-ai-capabilities-and-the-risk-of-bioterrorism-converting-capability-evaluations-to-risk-assessments (Accessed February 16, 2026).

[ref50] Rivera S. Hanke M. S. Curtis S. Cherian N. Gitter A. Gray J. J. . (2025). Responsible Biodesign Workshop: AI, Protein Design, and the Biosecurity Landscape – Recommended Actions. OSF Preprints. doi: 10.31219/osf.io/yq48e_v2

[ref51] Sengupta A. Yassif J. M. Severance H. (2024). International Bio Funders Compact. Washington: The Nuclear Threat Initiative.

[ref52] Smith A. A. Wong E. L. Donovan R. C. Chapman B. A. Harry R. Tirandazi P. . (2026). Using a GPT-5-driven autonomous lab to optimize the cost and titer of cell-free protein synthesis. 2026.02.05.703998. doi: 10.64898/2026.02.05.703998

[ref53] Tang Z. Somia N. Yu Y. Koo P. K. (2025). Evaluating the representational power of pre-trained DNA language models for regulatory genomics. Genome Biol. 26:203. doi: 10.1186/s13059-025-03674-8, 40660356 PMC12261763

[ref54] Thadani N. N. Gurev S. Notin P. Youssef N. Rollins N. J. Ritter D. . (2023). Learning from prepandemic data to forecast viral escape. Nature 622, 818–825. doi: 10.1038/s41586-023-06617-0, 37821700 PMC10599991

[ref55] The White House OSTP (2024). United States Government Policy for Oversight of Dual Use Research of Concern and Pathogens with Enhanced Pandemic Potential. Washington: The White House.

[ref56] The White House OSTP (2025). Executive Order: Improving the Safety and Security of Biological Research. Washington: The White House.

[ref57] UKRI (2025). Statement on Research with Potential Misuse Risk. Swindon: UK Research and Innovation.

[ref58] Wang D. Huot M. Zhang Z. Jiang K. Shakhnovich E. I. Esvelt K. M. (2026). Without safeguards, AI-biology integration risks accelerating future pandemics. Front. Microbiol. 16:1734561. doi: 10.3389/fmicb.2025.1734561, 41658008 PMC12872745

[ref59] Wang M. Zhang Z. Bedi A. S. Velasquez A. Guerra S. Lin-Gibson S. . (2025). A call for built-in biosecurity safeguards for generative AI tools. Nat. Biotechnol. 43, 845–847. doi: 10.1038/s41587-025-02650-8, 40295784

[ref60] Webster T. Moulange R. Del Castello B. Walker J. Zakaria S. Nelson C. (2025). Global Risk Index for AI-Enabled Biological Tools. The Centre for Long-Term Resilience website (2025). doi: 10.71172/wjyw-6dyc

[ref61] Wei B. Che Z. Li N. Sehwag U. M. Götting J. Nedungadi S. (2025). Best practices for biorisk evaluations on open-weight bio-foundation models. arXiv.org. Available online at: https://arxiv.org/abs/2510.27629v4 (Accessed March 13, 2026).

[ref62] Williams A. E. Del Castello B. Lee J. Roberts D. Tarangelo J. P. Atanda J. . (2026). Developing a risk-scoring tool for artificial intelligence–enabled biological design: a method to assess the risks of using artificial intelligence to modify select viral capabilities. Available online at: https://www.rand.org/pubs/research_reports/RRA4490-1.html (Accessed March 13, 2026).

[ref63] Williams A. E. Tarangelo J. P. Del Castello B. Moritz R. Popescu S. Sharkey M. (2025). Evaluating Safety and Security in Biological Research: A Proposed Tool to Assess Dual-Use Research of Concern and Pathogens with Enhanced Pandemic Potential. Available online at: https://www.rand.org/pubs/perspectives/PEA4125-1.html (Accessed March 13, 2026).

[ref64] Wittmann B. J. Alexanian T. Bartling C. Beal J. Clore A. Diggans J. . (2025). Strengthening nucleic acid biosecurity screening against generative protein design tools. Science 390, 82–87. doi: 10.1126/science.adu8578, 41037625

[ref65] World Health Organization (2022). Global Guidance Framework for the Responsible use of the life Sciences: Mitigating Biorisks and Governing dual-use Research. Available online at: https://www.who.int/publications/i/item/9789240056107 (Accessed March 13, 2026).

[ref66] World Health Organization (2024). WHO Launches a mobile app for Biosafety risk Assessment. Available online at: https://www.who.int/news/item/07-03-2024-who-launches-a-mobile-app-for-biosafety-risk-assessment (Accessed March 13, 2026).

[ref67] Yassif J. Severance H. Sengupta A. Samani H. Cameron E. E. Franz A. . (2025). Guidance for Assessing Biosecurity and Biosafety Risks: For the Purpose of Reviewing Research Proposals Prior to Funding. Washington: The Nuclear Threat Initiative.

[ref68] Zhang Z. Chakraborty S. Bedi A. S. Mathew E. Saravanan V. Cong L. . (2025a). Generative AI for biosciences: emerging threats and roadmap to biosecurity. doi: 10.48550/arXiv.2510.15975

[ref69] Zhang Z. Zhou Z. Jin R. Cong L. Wang M. (2025b). Gene breaker: jailbreak attacks against DNA language models with pathogenicity guidance. doi: 10.48550/arXiv.2505.23839

